# A Role for Alström Syndrome Protein, Alms1, in Kidney Ciliogenesis and Cellular Quiescence 

**DOI:** 10.1371/journal.pgen.0030008

**Published:** 2007-01-05

**Authors:** Guochun Li, Raquel Vega, Keats Nelms, Nicholas Gekakis, Christopher Goodnow, Peter McNamara, Hua Wu, Nancy A Hong, Richard Glynne

**Affiliations:** 1 Genomics Institute of the Novartis Research Foundation, San Diego, California, United States of America; 2 Phenomix Australia, Acton, Australia; 3 Australian Phenomics Facility, Australian National University, Canberra, Australia; 4 John Curtin School of Medical Research, Australian National University, Canberra, Australia; 5 Phenomix Corporation, San Diego, California, United States of America; 6 Novartis Institutes for BioMedical Research Incorporated, Cambridge, Massachusetts, United States of America; Stanford University School of Medicine, United States of America

## Abstract

Premature truncation alleles in the *ALMS1* gene are a frequent cause of human Alström syndrome. Alström syndrome is a rare disorder characterized by early obesity and sensory impairment, symptoms shared with other genetic diseases affecting proteins of the primary cilium. ALMS1 localizes to centrosomes and ciliary basal bodies, but truncation mutations in *Alms1*/*ALMS1* do not preclude formation of cilia. Here, we show that in vitro knockdown of *Alms1* in mice causes stunted cilia on kidney epithelial cells and prevents these cells from increasing calcium influx in response to mechanical stimuli. The stunted-cilium phenotype can be rescued with a 5′ fragment of the *Alms1* cDNA, which resembles disease-associated alleles. In a mouse model of Alström syndrome, Alms1 protein can be stably expressed from the mutant allele and is required for cilia formation in primary cells. Aged mice developed specific loss of cilia from the kidney proximal tubules, which is associated with foci of apoptosis or proliferation. As renal failure is a common cause of mortality in Alström syndrome patients, we conclude that this disease should be considered as a further example of the class of renal ciliopathies: wild-type or mutant alleles of the Alström syndrome gene can support normal kidney ciliogenesis in vitro and in vivo, but mutant alleles are associated with age-dependent loss of kidney primary cilia.

## Author Summary

Alström syndrome is a rare genetic disorder caused by mutations in the *ALMS1* gene. The disease is characterized by blindness, deafness, and metabolic disorders. These symptoms are reminiscent of diseases affecting the primary cilium, a cellular appendage with a role in sensing changes to the extracellular environment. In addition, kidney failure is a frequent cause of death in Alström syndrome patients, and recent studies have suggested a causal relationship between defects in primary cilia and cystic kidney diseases. In this paper, we show that ALMS1 protein is required to form cilia in kidney cells. Mutant alleles of the gene that are similar to those seen in the human disease are able to support cilia formation in cell culture and in animals. However, a defect in the function of the disease alleles is uncovered in older mice: cilia are lost from the kidney cells, and this is associated with an increase in cellular proliferation and cell death. The data are consistent with a requirement for ALMS1 in ciliogenesis and suggest inclusion of Alström syndrome among the growing class of cilia-related pathologies.

## Introduction

Alström syndrome is a rare autosomal recessive disorder characterized by early onset obesity, type 2 diabetes mellitus, retinal degeneration, and hearing impairment. Other aspects of the disease include cardiomyopathy, liver dysfunction, kidney dysfunction, and a delay in puberty. Renal function declines with age, and end-stage renal disease is a common cause of death in Alström syndrome patients [1]. Additionally, enlarged kidneys have been reported in a previously reported mouse strain with a mutation in *Alms1* [2].

The primary cilium is an antenna-like organelle, surrounded by a membrane contiguous with the plasma membrane [3,4]. Typically, cilia extend several micrometers from the apical face of the cell, grounded to the cellular microtubule complex through the basal body. Cilia are conserved through several eukaryotic phyla, including *Caenorhabditis elegans, Chlamydomonas,* and vertebrates. Immotile primary cilia contain a scaffold of nine microtubule doublets running the length of the axoneme (9 + 0), whereas motile cilia contain an additional central microtubule pair (9 + 2 arrangement). Increasing data demonstrate roles for the primary cilium in sensory functions. These include mechanosensation of lumenal flow in kidney tubules and transduction of extracellular signaling through the hedgehog, Wnt, and platelet-derived growth factor receptor pathways [3,4].

Four lines of evidence suggest the hypothesis that renal failure in Alström patients is secondary to a defect in primary cilia in the kidney. First, mutations in genes that are implicated in the function of primary cilia are associated with kidney diseases. Polycystic kidney disease (PKD) is characterized by progressive development of fluid-filled cysts, ultimately leading to end-stage renal failure [5]. Both autosomal dominant PKD (ADPDK) proteins (Polycystin-1 and −2) are localized to primary cilia and are necessary for cilia-mediated signaling in response to a fluid-flow stimulus [6]. Autosomal recessive PKD (ARPKD) protein (fibrocystin) and nephronophthisis disease proteins, nephrocystin and inversin, are involved in ciliary protein transport [7,8]. Additionally, mouse strains with genetic lesions in ciliary proteins lead to cystic kidney disease [9,10]. Second, the spectrum of phenotypes seen in Alström patients is similar to Bardet-Biedl patients [11], suggesting that mutations in *ALMS1* might cause disease through a similar mechanism to *BBS* mutations. It is now well documented that several of the 11 genes mutated in Bardet-Biedl syndrome (BBS) have roles in the function of primary cilia [12–19]. Third, ALMS1 is localized to centrosome and ciliary basal bodies in vitro [20,21], consistent with a role in the structure of the basal body or in the transport of proteins between the cytoplasm and the ciliary axoneme. Fourth, in vivo phenotypes of *Alms1* mutant mice include a lack of sperm flagella, a modified ciliary structure, as well as defective rhodopsin transport through the connecting cilia of photoreceptor cells [2,22].

These data point to a role of ALMS1 in the function of primary cilia, but no defects in cilia formation were observed in human dermal fibroblasts from an Alström syndrome patient [21] or in the kidney collecting duct epithelial cells of a gene-trap *Alms1* mutant mouse strain [2]. A possible resolution of this discrepancy comes from the genetic analysis of mutations in *ALMS1* that have been found in patients. In all cases, there was at least one allele of *ALMS1* that could encode an N-terminal fragment of the ALMS1 protein before the presence of a premature stop codon. Moreover, the alleles in two reported mice models of Alström syndrome would also be predicted to encode the N-terminus of Alms1 [2,22]. Thus, it is possible that ALMS1 is required for cilia formation, and that the disease-associated alleles are able to provide at least part of this activity through expression of the N-terminus of the protein. In this study, we test whether ALMS1 has a role in cilia formation or function and provide evidence that renal failure in Alström syndrome patients might be associated with ciliary dysfunction.

## Results

### Depletion of Alms1 mRNA and Protein by Short Interfering RNA Causes Defective Ciliogenesis in Kidney Cells

**Figure 1:**
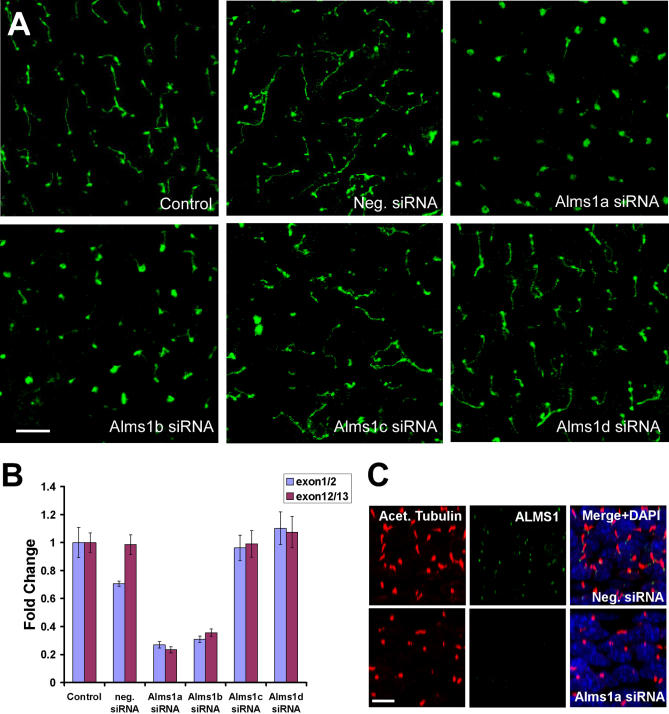
Suppression of *Alms1* Expression Alters Primary Cilium Formation in Kidney Epithelial Cells (A) Elongated cilia, visualized with staining of acetylated tubulin (green), form normally in mIMCD3 cells after mock-transfection, transfection with a negative control siRNA, or transfection with two inactive siRNAs directed against Alms1 (Alms1c and Alms1d). Focal staining of acetylated tubulin without axoneme extension is seen after transfection with two active siRNAs targeting *Alms1* (Alms1a and Alms1b). (B) Real-time PCR analysis with two mouse *Alms1* probes recognizing the junctions of exons 1 and 2 and exons 12 and 13, respectively: Alms1a and Alms1b siRNAs both cause 70%–80% knockdown of *Alms1* mRNA; no effect on *Alms1* mRNA was seen with the three siRNAs that were inactive in the ciliogenesis assay. (C) Alms1a siRNA-treated cells lose endogenous Alms1 protein expression. Acet, acetylated. Scale bars, 10 μm.

To determine whether Alms1 was necessary for cilium formation and/or function, we used an in vitro model of kidney cell ciliogenesis and signaling. Mouse inner medullary collecting duct (mIMCD3) cells form cilia 5 d after confluency [23]. The cilia can be visualized as long protrusions from the cell surface using an antibody raised against acetylated tubulin (Figure 1A). We tested several short interfering RNA (siRNA) molecules designed against the mouse *Alms1* sequence for their effects on formation of cilia in this model. Cilia were formed normally in the presence of a negative control siRNA. However, transfection with two siRNA sequences against *Alms1,* Alms1a and Alms1b, led to a markedly different phenotype. The acetylated tubulin staining on each cell manifested as a single ball of fluorescence, and very few cells showed the elongated staining typical of ciliary axoneme (Figure 1A). Both of these siRNA species also caused a decrease in the *Alms1* mRNA level (Figure 1B). In contrast, two additional siRNAs (Alms1c and Alms1d) did not affect the pattern of acetylated tubulin staining in the ciliogenesis assay, nor did they affect the *Alms1* mRNA level (Figure 1A and 1B). To demonstrate that the active siRNA species also decreased levels of Alms1 protein, we raised an antiserum in rabbits against the predicted N-terminal 13 amino acids of the open reading frame of *Alms1.* Using this antiserum, we detected positive staining at the base of cilia, consistent with previous reports on the subcellular localization of ALMS1 [21]. The antibody signal was reduced below detection in the presence of the active siRNA molecules (Figure 1C).

### Loss of Alms1 Does Not Affect Transcriptional Regulation of Ciliary Genes but Does Disrupt Ciliary Mechanosensation

**Figure 2:**
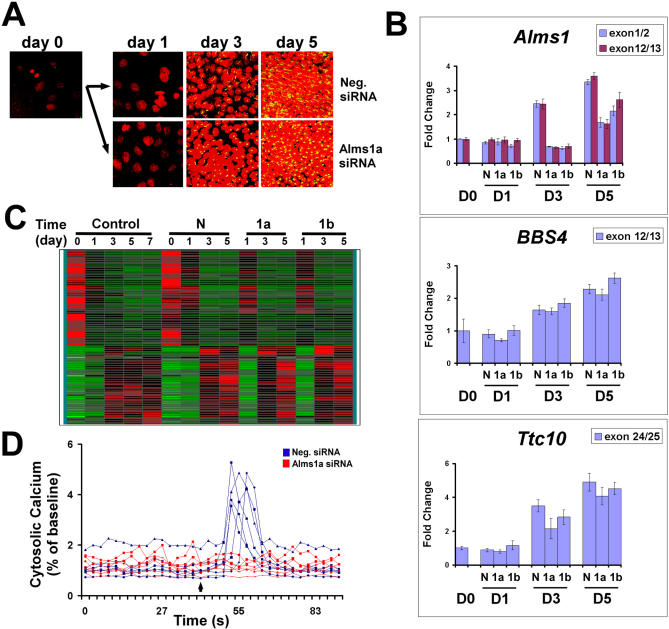
Loss of Alms1 Does Not Affect Transcriptional Changes during Ciliogenesis but Causes Impairment in Flow-Induced Mechanosensation (A) Confocal microscopic analysis of cilia biogenesis in mIMCD3 cells. Short cilia can be detected at day 3 after transfection. mIMCD3 cells transfected with Alms1a siRNA have stunted cilia at days 3 and 5. Cells were stained with anti-acetylated tubulin (yellow, cilia) and TO-PRO-3 (red, nuclei). (B) Suppression of Alms1 does not affect the upregulation of *Bbs4* and *Ttc10* during ciliogenesis. N, negative siRNA; 1a, Alms1a siRNA; 1b, Alms1b siRNA. (C**)** Heat map representation of microarray analysis of mIMCD3 during ciliogenesis. 98 genes with the most dramatic changes in expression showed approximately equivalent expression changes in the presence of a scrambled siRNA control, or in the presence of specific siRNAs that decreased *Alms1* mRNA levels and blocked cilia formation. (D) Stunted cilia formed in the presence of Alms1a siRNA (red) lack flow-induced Ca2+ influx in mIMCD3 cells, compared with a negative control siRNA (blue). Representative data are shown for cytosolic calcium change of individual cells in response to mechanical flow. Arrow points to the start of flow.

Transcriptional profiling in *Chlamydomonas* and in C. elegans has identified a set of genes that are regulated during ciliogenesis [24,25]. As an initial characterization of the role of Alms1 in the mIMCD3 in vitro model, we asked whether Alms1 protein was required for the transcriptional response associated with ciliogenesis. A time course of mIMCD3 ciliogenesis showed a steady rise in the level of *Alms1* mRNA after confluency. This was paralleled by an increase in cilia length (Figure 2A and 2B). We chose two other cilia-related genes that have increased mRNA levels during ciliogenesis, *Bbs4* and *Ttc10. Bbs4* (BBS gene 4) encodes a protein localized to the basal body and centrosome that is required for targeting cargo to the pericentriolar region [13]; *Ttc10* (IFT88/polaris) is required for transport of cargo from basal body to the distal cilium [26]. *Bbs4* and *Ttc10* mRNA were upregulated even when ciliogenesis was disrupted with Alms1a siRNA (Figure 2B). Moreover, microarray analysis showed that knockdown of *Alms1* by siRNA had no effect on the general transcription program associated with confluency and ciliogenesis in the mIMCD3 cells (Figure 2C and Table S1 **)**.

We then used this model to ask whether the stunted cilia formed in limiting amounts of Alms1 were functionally competent. Cilia on kidney epithelial cells are able to induce an intracellular calcium signal in response to fluid flow over the cell surface. Defects in this response are thought to underlie PKD [27,28]. Full-length cilia formed in the presence of a control siRNA were able to respond to flow as expected (Figure 2D). However, the stunted cilia formed in the presence of the Alms1a siRNA were unable to induce calcium flux after a flow stimulus.

Taken together, these in vitro results suggest a critical role of Alms1 in cilia biogenesis of kidney epithelial cells, though without disruption of the transcriptional program that accompanies this process. The abnormal cilia formed after knockdown of *Alms1* are characterized by a focal concentration of acetylated tubulin at the cell membrane, and these stunted cilia are unable to respond to fluid flow by calcium flux.

### A 5′ Fragment of Alms1 Is Sufficient to Rescue Cilia Formation In Vitro after Knockdown of Endogenous Alms1 and Can Support Normal Ciliogenesis In Vivo

**Figure 3:**
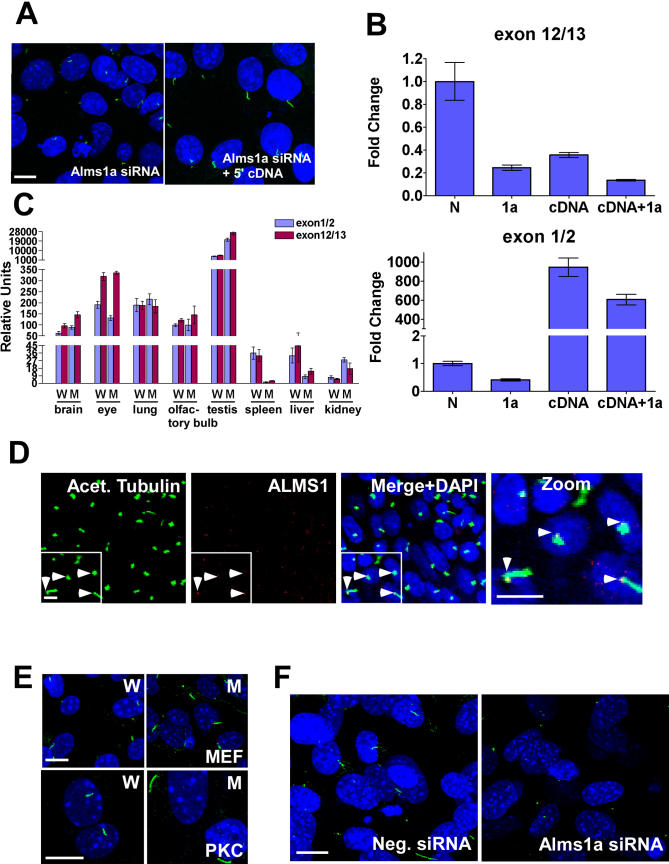
N-Terminal Alms1 Protein Can Support Cilia Formation (A) Cotransfection of Alms1a siRNA-treated cells with a 5′ *Alms1* cDNA construct rescues primary cilia formation in mIMCD3 cells. (B) Real-time PCR analysis of Alms1a siRNA and N-terminal Alms1-transfected cells. Upper panel: over-expression of the 5′ cDNA does not affect knockdown of endogenous *Alms1* mRNA with Alms1a siRNA. Lower panel: knockdown of endogenous *Alms1* mRNA does not affect overexpression of the 5′ cDNA. N, negative control siRNA; cDNA, 5′ *Alms1* cDNA; 1a, Alms1a siRNA. (C) Stable expression of *Alms1* mRNA from the *Alms1^L2131X/L2131X^* allele. Real-time PCR analysis of *Alms1* gene expression in an *Alms1^L2131X/L2131X^* mouse and a wild-type littermate control. (D) The N-terminal mouse Alms1 antibody detects Alms1 mutant protein at the ciliary basal body in primary kidney cells from the *Alms1^L2131X/L2131X^* mouse. Shown are low and high magnifications of the ciliated cells. Arrowheads point out the base of cilia. (E) Normal appearance of primary cilia in primary fibroblasts (MEF) and primary kidney cells (PKC) from the *Alms1^L2131X/L2131X^* mouse strain. (F) Inhibition of cilia formation in Alms1a siRNA-treated *Alms1^L2131X/L2131X^* primary fibroblasts. Scale bars, 10 μm.

Reported alleles of *ALMS1* in human patients, as well as in two reported mouse models of the disease, usually encode premature termination codons in exons 8, 10, and 16 [2,22,29,30]. In these alleles, the 5′ terminus of the gene encodes an intact open reading frame that might give rise to a partially functional protein. Therefore, we tested whether a truncated 5′ cDNA was able to rescue cilia formation after *Alms1* knockdown. We transfected a mouse *Alms1* cDNA encoding the N-terminal 1,282 amino acids, equivalent to exons 1–8, into mIMCD3 cells in the presence of the Alms1a siRNA. The Alms1a siRNA sequence matches the *Alms1* gene 3′ of the cDNA fragment and so was predicted to affect only the levels of the endogenous transcript and not expression of the cDNA encoding the N-terminus of Alms1. As expected, transfection with Alms1a siRNA caused focal acetylated tubulin staining. However, cotransfection of the *Alms1* cDNA with Alms1a siRNA was fully capable of rescuing the cilia phenotype (Figure 3A). To monitor the effects of cDNA transfection on knockdown of the endogenous mRNA level, we used a Taqman assay recognizing the boundary of exons 12 and 13 of the *Alms1* mRNA. This assay will detect the endogenous full-length transcript but not the transfected cDNA and shows that cotransfection of the cDNA did not affect knockdown of the endogenous transcript. Conversely, using a Taqman assay recognizing the 5′ of *Alms1* mRNA that detects both the endogenous and cDNA-encoded transcript, we showed that cotransfection with the siRNA Alms1a did not affect over-expression of the *Alms1* 5′ cDNA (Figure 3B).

To determine whether a premature truncation allele of the *Alms1* gene can support normal ciliogenesis at endogenous expression levels, we used primary cells from a mouse strain with an ethyl nitroso urea–induced, premature truncation mutation in exon 10 of the *Alms1* gene (Figure S1). The *Alms1* gene in this mutant strain is predicted to encode the N-terminal 2,131 amino acids of the mouse Alms1 protein, and we therefore named the allele *Alms1^L2131X/L2131X^.* Similar expression of *Alms1* mRNA was observed across eight tissues in wild-type and homozygous *Alms1^L2131X/L2131X^* mice (Figure 3C). In particular, levels of *Alms1* mRNA in the kidney of mutant *Alms1^L2131X/L2131X^* mice are at least as high as in the wild-type control mice. In addition, we detected mutant Alms1 protein localized at the base of cilia of *Alms1^L2131X/L2131X^* primary kidney cells (Figure 3D). Thus, despite the presence of a premature stop codon allele in the *Alms1* gene of these mice, both mRNA and protein were readily detectable. By isolating primary fibroblasts and kidney cells from *Alms1^L2131X/L2131X^* mice and wild-type controls, we found that cells from the *Alms1^L2131X/L2131X^* strain showed normal primary cilia when compared to wild-type cells (Figure 3E), which is consistent with previous reports [2,21]. We then tested whether expression of the mutant *Alms1* transcript was required for normal cilia formation in cells derived from the *Alms1^L2131X/L2131X^* mouse strain. Knockdown of mutant *Alms1* mRNA using a 5′ specific siRNA in the *Alms1^L2131X/L2131X^* embryonic fibroblasts inhibited ciliogenesis in these cells (Figure 3F), as was seen for wild-type cells (unpublished data).

### Age-Dependent Loss of Primary Cilia and Homeostasis in Alms1 Mutant Mice

**Figure 4:**
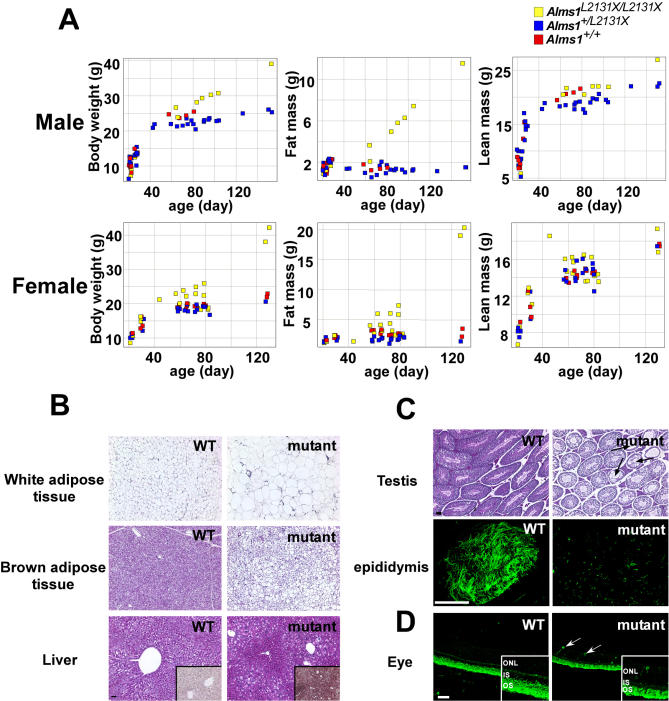
*Alms1^L2131X/L2131X^* Mice Recapitulate Human Alström Syndrome (A) *Alms1^L2131X/L2131X^* mice gain more fat mass than heterozygote or wild-type controls but equivalent lean mass. (B) Histological examination of *Alms1^L2131X/L2131X^* mice and wild-type littermate control. Insets show oil red O staining of frozen liver sections. (C) Testis H&E sections show degeneration of seminiferous tubules (arrow), which have reduced numbers of germinal cells. Reduced numbers of sperm flagella with decreased length are observed in the epididymus of *Alms1^L2131X/L2131X^* mice (anti-acetylated tubulin, green). H&E, hematoxylin-eosin. (D) Rhodopsin staining in the outer nuclear layer cell bodies is seen in rare cells in the *Alms1^L2131X/L2131X^* animals (arrows) but not in wild-type littermate controls. Insets illustrate higher magnification images. ONL, outer nuclear layer; IS, inner segment; OS; outer segment. Scale bars, 50 μm.

We characterized the phenotype of *Alms1^L2131X/L2131X^* mouse strain with reference to the metabolic and sensory defects reported in Alström syndrome patients to determine whether the pathology of the mouse strain resembled that of human patients. Homozygous mutant mice increased in weight faster than wild-type controls between 7 and 10 wk of age, and body composition analysis showed that this increase was almost entirely explained by an increase in fat mass (Figure 4A). Histological analysis showed hypertrophy of white and brown fat adipocytes and steatosis of the liver in obese mutant mice (Figure 4B). Although one older male mutant mouse in this cohort of 20 became diabetic, most mice had normal glucose levels with hyperinsulinemia. Other serum abnormalities include elevated leptin, triglycerides, total cholesterol, and HDL cholesterol (Figure S2). Finally, we noted that the *Alms1^L2131X/L2131X^* strain had defective sperm formation in the testes and defective rhodopsin transport in the retina (Figure 4C and 4D). We conclude from these data that the *Alms1^L2131X/L2131X^* mouse strain is a close genetic model of human Alström syndrome [1], and we proceeded to analyze whether there were any defects in the kidney cilia that might relate to renal failure in human patients.

**Figure 5:**
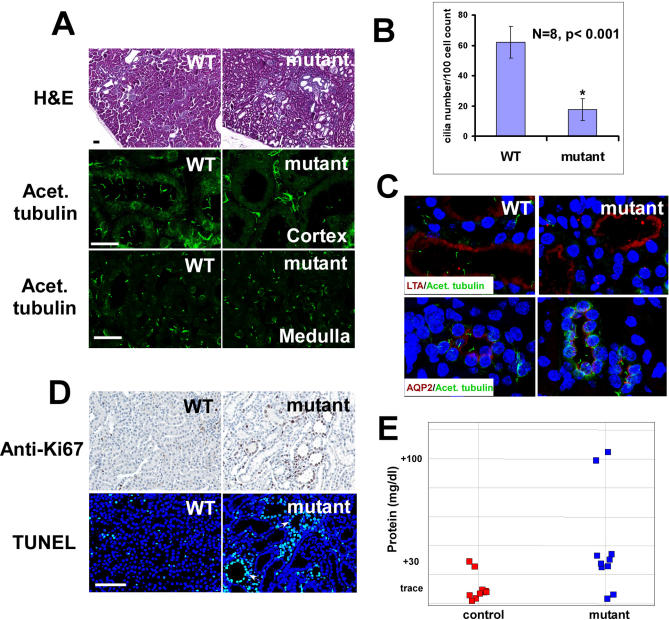
Kidney Abnormalities in *Alms1* Mutant Mice (A) H&E-stained kidney sections of a 6-mo-old *Alms1^L2131X/L2131X^* mouse showing dilated cortex tubules compared with an age-matched wild-type control. Lack of kidney cilia is observed in some tubules in the cortex of *Alms1^L2131X/L2131X^* kidney, whereas cilia in the medulla appear normal. H&E, hematoxylin-eosin; Acet, acetylated. (B) Cortex cilia count comparison between *Alms1^L2131X/L2131X^* and controls. 300–400 kidney nuclei were examined for each of six fields of *Alms1^L2131X/L2131X^* and wild-type controls. The bar chart represents the average and standard deviations of cilia count per 100 kidney cortex epithelial cells from eight mice per group. (C) In the cortex of *Alms1^L2131X/L2131X^* kidney, cilia are lost selectively in LTA-labeled tubules but not in aquaporin-2-expressing tubules. (D) Upper panel: clusters of Ki67-positive proliferating epithelial cells in the *Alms1^L2131X/L2131X^* kidney, potentially lining the same convoluted tubule. Lower panel: TUNEL staining reveals apoptotic cells in *Alms1^L2131X/L2131X^* kidneys but rarely in a wild-type control. Arrow, whole tubule cross sections were labeled by TUNEL, suggesting progression of nephropathy in *Alms1^L2131X/L2131X^* mutant kidneys. WT, wild-type. (E) Urinalysis of 3- to 6-mo-old *Alms1^L2131X/L2131X^* mice and age-matched littermate controls. Urine from *Alms1^L2131X/L2131X^* mice showed slight elevation of protein levels, *p* = 0.007. Scale bars, 50 μm.

The kidneys of 6-mo-old *Alms1^L2131X/L2131X^* mice contained multiple dilated tubules in the cortex (Figure 5A). Consistent with a previous report [2], primary cilia appeared normal in collecting ducts of the renal medulla. However, we noticed that some cortex tubules appeared to be almost completely denuded of cilia in aged *Alms1^L2131X/L2131X^* mutants (Figure 5A and 5B). To further characterize these tubules, we stained the kidney sections with Lotus tetragonolobus agglutinin (LTA) as a proximal tubular marker, and with aquaporin-2 antibody as a marker of collecting ducts (Figure 5C). In both the wild-type and *Alms1^L2131X/L2131X^* mutant animals, the aquaporin-2-labeled collecting duct cells have clear primary cilia as expected. LTA-labeled proximal tubule cells in the wild-type animals were also ciliated. In contrast, most of the LTA-labeled proximal tubules of *Alms1^L2131X/L2131X^* mutants were not ciliated.

Human PKD is thought to originate from renal tubule dilatation, secondary to abnormalities in cellular proliferation and apoptosis. We examined kidney sections from wild-type and mutant mice with a marker for proliferation, Ki67, and by TUNEL staining for apoptotic cells (Figure 5D). Sparse proliferation was seen in wild-type kidney sections. Higher levels of proliferation were detected in the cortex of kidneys from *Alms1^L2131X/L2131X^* mice, and this was focused in patches, perhaps representing convolutions of the same tubule coming into the plane of sectioning. In most of the cross sections of dilated tubules, several (20%–50%) of the epithelial cells were Ki67-positive, and these cells lacked cilia. We also noted a dramatic increase in kidney epithelial cell apoptosis in the mutant mice. As with Ki67, it was striking that TUNEL-positive cells had a nonuniform distribution and seemed to be clustered within particular tubules, whereas other tubules appeared identical to wild-type controls. These changes appeared to compromise kidney function, as urinanalysis revealed a mild proteinurea in adult *Alms1^L2131X/L2131X^* mice (Figure 5E). None of the kidney phenotypes described above (dilated tubules, loss of cilia, proliferation and apoptosis of epithelial cells, or proteinurea) was seen in 2-mo-old mice (Figure S3 and unpublished data).

## Discussion

*ALMS1* is among the largest disease genes identified today in the human genome. However, amino acid sequence analysis identifies only a leucine zipper near the N-terminus, allowing limited inferences to be made about the function of this protein. Localization of ALMS1 to the centrosome and ciliary basal body has been determined using a green fluorescent protein fusion to its C-terminus, and with an N-terminal antibody, respectively [20,21]. These data are consistent with a role for ALMS1 in the function of cilia, which is supported by the overlapping clinical phenotypes between Alström syndrome and other ciliopathies, especially BBS. Additionally, mouse models of BBS and Alström syndrome lack sperm flagella and a modified cilium; and show aberrant transport of rhodopsin, pointing to defective function of the connecting cilium in photoreceptor cells.

In this paper, we investigated whether Alms1 protein is required for the formation of cilia and, if so, whether cilia can be formed in human or mouse cells with a mutated *ALMS1/Alms1* gene. We used an in vitro model of kidney ciliogenesis to demonstrate that knockdown of *Alms1* mRNA caused a striking alteration in the morphology of cilia: knockdown of *Alms1* caused stunted or focal staining of acetylated tubulin. The specificity of the siRNA reagents was confirmed by two pieces of evidence. First, two siRNAs that were active in decreasing mRNA expression were also active in decreasing formation of elongated cilia. Conversely, two additional *Alms1* siRNAs, as well as a negative control siRNA, did not decrease *Alms1* mRNA nor did they affect the ciliary phenotype. Second, the ciliary phenotype that was induced by *Alms1* siRNA knockdown could be rescued by cotransfection of a 5′ fragment of *Alms1* that was not targeted by the active siRNA. Together these results rule out the possibility that the cilia phenotype caused by siRNA knockdown was an off-target effect, and point to a role of Alms1 in the maintenance or biogenesis of cilia.

Interestingly, previous work had shown that cilia were formed in cells with mutant *ALMS1/Alms1.* To reconcile those findings with our siRNA knockdown data, we posited a functional role for the truncated Alms1 protein encoded by the mutant alleles. *Alms1* mRNA in our mutant mouse strain was present at levels similar to those in wild-type mice, despite the presence of a premature stop codon. We also found that the mutant Alms1 protein was detectable and localized to ciliary basal bodies in cells from these mice, just as was observed for wild-type Alms1. Additionally, in unpublished data, immunohistochemistry with the antibody raised against the N-terminus of Alms1 showed prominent staining in the mutant pancreatic islets, confirming that protein could be stably expressed from the mutant allele. To test whether there was a function for the mutant protein, we showed that knockdown of the mutant *Alms1* mRNA inhibited ciliogenesis in primary cells from these mice. We also showed that the ciliary phenotype induced by knockdown of *Alms1* could be rescued using a cDNA encoding only the N-terminal 1,282 amino acids of Alms1, confirming that the N-terminus of Alms1 is sufficient to support cilia formation.

A spectrum of nonsense and frameshift mutations cause Alström syndrome, and all have different effects. However, no genotype/phenotype correlation has been observed among human patients [29–31]. Furthermore, two mouse models of Alström syndrome reported to date, as well as a third model reported in this manuscript, contained alleles that were predicted to encode the N-terminus of the protein, and the reported phenotypes are very consistent. Thus, we suggest that truncation of ALMS1/ Alms1, secondary to mutations in (predominantly) exons 8, 10, and 16, leads to similar phenotypes in human and mouse. In particular, we provide evidence for residual function of the disease-associated alleles. This residual function of mutant *Alms1* alleles might explain a lack of the more severe developmental phenotypes described in animal models with mutations of intraflagellar transport (IFT) or kinesin motor proteins [26,32,33].

There is growing support for the “antenna” role of primary cilium as a key participant in sensing environmental stimuli, transduction of intracellular signaling, and regulation of morphogenetic pathways. Studies of BBS patients and BBS knockout animal models have revealed the role of primary cilia in sensing of light and olfaction [14,16,17,19]. The kidney primary cilium, extending from the tubular epithelium into the lumen, has been implicated in mechanosensation: genetic or chemical disruption of cilia inhibited intracellular calcium influx in response to laminar flow [34,35]. Recent studies have suggested that cilia constitute an essential platform where signaling processes are initiated. The Hedgehog receptor Smoothened and the platelet-derived growth factor receptor alpha localize to the cilium and, in both cases, ciliary localization is required for signaling transduction [36,37]. We show here that Alms1 is necessary for formation of kidney epithelial cilia, which in turn are known to be essential for mechanosensory signaling.

Well-characterized genetic disorders of primary cilia include the family of PKD, inherited either as ADPKD or ARPKD traits. While ARPKD is congenital and often causes fetal or neonatal death, the onset of the ADPKD is mainly in adulthood with age-dependent progression [5]. Animal models of PKD have been developed by genetic mutation of PKD genes [38–41], loss of kidney cilia by inhibition of IFT components [10,42], or constitutive activation of the Wnt pathway [43].

Recent data have started to build a connection between these seemingly unrelated genetic perturbations in PKD, IFT, and Wnt genes. Localization of PKD proteins has been observed on, or at the base of, cilia and cilia are dependent on IFT for protein transport to and from the cytoplasm [44]. Both PKD1 and PKD2 function on cilia to sense lumenal flow and control cell proliferation. Specifically, fluid flow over the cilia increases expression of inversin, which in turn targets cytoplasmic Dsh, inhibiting the canonical Wnt pathway [45]. It has been proposed that urine flow terminates the canonical Wnt pathway in favor of the noncanonical Wnt pathway. The noncanonical Wnt pathway might then maintain planar cell polarity and restrict cell division in a direction parallel to the long axis of the tubule. Moreover, BBS proteins have been shown to directly interact with proteins which regulate planar cell polarity [46], as well as in ciliary protein transport [47], suggesting a model in which defects in cilia disrupt planar cell polarity signaling and lead to disorientated cell division and cyst formation.

Existing mouse PKD animal models are characterized by the formation of early-onset cysts in the collecting tubules, reminiscent of human ARPKD. In contrast, the *Alms1* mutant mouse model presented here shows cilia loss in the proximal tubules. Further, adult mice progress to a breakdown in maintenance of kidney epithelial cellular quiescence, with a dramatic increase in apoptotic and proliferating cells restricted to those tubules that have lost cilia. This is accompanied by tubular dilatation and mild proteinurea in older mutant mice. All these phenotypes are similar to clinical features of ADPKD, making the *Alms1^L2131X/L2131X^* mouse strain an interesting animal model to study the relation of cilia loss, altered signaling, and cellular proliferation in the progression of cystic kidney disease in the proximal tubules.

Initiation of ADPKD is suggested to be dependent on a second somatic mutation in the wild-type allele of PKD1 or PKD2. Evidence for this hypothesis is controversial as somatic mutagenesis rates in nontransformed cells are likely to be too low to explain the high prevalence of bilateral disease in heterozygous carriers [48,49] and a reduced expression of PKD1 is sufficient to initiate cystogenesis [41]. In this recessive model of ciliary dysfunction in the kidney, we also observe localized expression of the cellular phenotype (cilia loss, apoptosis, or proliferation). This suggests that in this model, and perhaps in human late-onset kidney diseases, non-genetic and/or epigenetic factors are necessary for expression of the phenotype. The disease-like allele of *Alms1,* although functionally able to support cilia formation in vitro, in young mice and in older mice in the kidney medulla, might reveal a compromised function in the cortex during aging. We also note that the expression of the cellular kidney phenotype in our model is not random but shows a variegated pattern on a tubule-by-tubule basis. Some tubule cross sections appear to have normal cilia, and little to no proliferation or apoptosis, whereas other tubule cross sections show extensive loss of cilia, sometimes with all or nearly all of the epithelial cells either proliferating or in apoptosis. The presumed somatic event, which, in combination with a genetic predisposition, causes some tubules to appear dysfunctional while neighboring tubules have a wild-type appearance, is not known. However, more detailed characterization of the progression of the phenotype might suggest treatments that maintain tubular integrity and delay disease.

## Materials and Methods

### Cell culture and ciliogenesis assay.

mIMCD3 cells were obtained from American Type Culture Collection (www.atcc.org) and cultured in DMEM/F12 media (Invitrogen, http://www.invitrogen.com) supplement with 10% FBS, 2.5 mM L-glutamine. For cilia formation assay, 50,000 cells were seeded on 12-mm Transwell filters (Costar). Filters were collected at day 0, day 1, day 3, day 5, and day 7 after seeding for RNA isolation and cilia immunostaining.

### Knockdown of *Alms1* expression by RNAi.

We designed siRNA specific to *Alms1* by online BLOCK-iT RNAi designer (Invitrogen). These siRNA sequences are: 5′-GCTGTATGTAGTCGAATTA-3′ (Alms1a); 5′-GCCTGATTCCTTGTTTCAA-3′ (Alms1b); 5′-GCAGTAGTCTCTTCTGCTT-3′ (Alms1c); 5′-GCTTCAGCTTTGCTGAATT-3′ (Alms1d). SiRNA were synthesized by Qiagen (http://www1.qiagen.com). For RNAi transfection, mIMCD3 cells were seeded as previously described and transfected with Lipofectamine 2000 (Invitrogen) according to manufacturer's instruction.

### DNA constructs.

The mouse *Alms1* cDNA was generated from mIMCD3 mRNA. N-terminus (aa: 1–1,282) was cloned into p3XFLAG-CMV vector (Sigma, http://www.sigmaaldrich.com) to generate N-Alms1-FLAG plasmid.

### RNA isolation and labeling for array analysis.

Cells were transfected on day 0 with either a scrambled siRNA control (one of two siRNAs directed against *Alms1*) or mock-transfected. Cells were seeded on day 1 and allowed to reach confluence through day 7. Cilia were formed in the mock-transfected and scrambled siRNA-transfected cells. Samples were taken for gene expression on days 0, 1, 3, 5, and 7 for the mock-transfected cells; days 0, 1, 3, and 5 for scrambled siRNA-transfected cells; and days 1, 3, and 5 for cells transfected with siRNAs against *Alms1.* RNA was isolated using the RNeasy Kit from Qiagen. Total RNA was examined on an Agilent Bioanalyzer (Agilent, http://www.agilent.com) and the 28S/18S ratio exceeded 2.0 for all samples. cDNA and cRNA were prepared from 4 μg total RNA according to standard Affymetrix protocols (http://www.affymetrix.com/support/technical/manual/expression_manual.affx). The in vitro transcription kit used was the GeneChip IVT labeling kit (Affymetrix). All reactions were processed at the same time by the same individual. We selected a group of 98 genes that had the highest relative gene expression change during ciliogenesis in the mock-transfected cells. The query was: (max expression level for days 0, 1, 3, 5, and 7 for the mock-transfected cells) divided by the maximum (the minimum expression level over these samples and a cutoff value of 400 arbitrary units). Four hundred units was approximately the 77th percentile of expression across all 36,000 probesets for a given sample. We selected the top 98 genes by this ratio (ratio >7.4) and then clustered the genes based on their expression levels in the mock-transfected samples. Comparison of this pattern with the samples that were transfected with either a scrambled or targeted siRNA showed no significant changes in the broad transcriptional program that was associated with ciliogenesis, suggesting that Alms1 is unlikely to play a role in transcription regulation associated with ciliogenesis.

### Ca^2+^ imaging assay.

mIMCD3 cells, grown on cover glass, were transfected with Alms1 RNAi or scrambled control siRNA using Lipofectamine 2000 (Invitrogen), and cells were grown for 5 more d to induce cilia formation. Ca^2+^ imaging was performed as described [6]. Briefly, cells were incubated with 5 μM Fura2-AM (Molecular Probes, Invitrogen) for 30–60 min at 37 °C. Cover glasses were placed in a laminar flow perfuse chamber (Warner Instruments Corporation, http://www.warneronline.com). After 20 min equilibration, cells were perfused with media as described [6] via a local perfusion pipette. Image of Fura-2 loaded cells with excitation wavelength alternating between 340 nm and 380 nm were captured by a CCD camera. Following subtraction of background fluorescence, the ratio of fluorescence at two wavelengths was analyzed using Metafluor (Universal Imaging Corporation). All experiments have been repeated in triplicate and similar results were obtained.

### Generation and phenotypic analysis of *Alms1* mutant mice.

We identified *Alms1^L2131X/L2131X^* mice in an ethyl nitroso urea–forward genetics screen. Mutant mice were generated by successive intercrossing of heterozygote *Alms1^L2131X^* animals on a mixed C57Bl6/ NOD genetic background. Body composition was determined by quantitative magnetic resonance according to protocols supplied by the manufacturer (Echo Medical Systems, http://www.echomri.com). All plasma analytes were measured in retinal blood samples. Serum glucose, cholesterol, HDL, and triglyceride levels were determined by Olympus AU400e. Leptin and insulin levels were analyzed by ELISA (Crystal Chemical Incorporated).

### Histology and immunohistochemistry.

Mice were anesthetized with Avertin (Sigma, 1.25%, 0.02 ml per gram body weight), perfused with 10% sucrose solution and 4% paraformaldehyde. Tissues were fixed in 4% paraformaldehyde, processed, and embedded in paraffin. 5-μm tissue sections were processed for hematoxylin-eosin (H&E) and immunostaining. For routine immunostaining, the sections were deparaffinized with xylene and rehydrated with graded ethanol. Primary antibodies used in this study were: γ-tubulin; acetylated tubulin (Sigma); β-Galactosidase (Abcam, http://www.abcam.com/index.html?); ki67 (NeoMarker Rabbit Monoclonal); aquaporin-2 (Santa Cruz Biotechnology, http://www.scbt.com). We generated polyclonal antibodies to Alms1 by injecting rabbits with the synthetic peptides MEPEDLPWPDELE, representing amino acids 1–13 of mouse Alms1. Dilutions were following the manufacturer's suggestions. Antibodies were visualized by Vectastain immunodetection kit (Vector Laboratories, Incorporated, http://www.vectorlabs.com). For indirect immunofloresence, Alexa488 and Texas Red conjugated secondary antibodies were obtained from Molecular Probes, Incorporated.

### Isolation and culturing primary fibroblasts and kidney cortical duct cells.

Wild-type and *Alms1^L2131X/L2131X^* embryos were harvested at embryonic day 12.5 and minced by passage through a syringe with an 18 G needle. Embryonic fibroblasts were cultured in 10% FBS DMEM medium. For generation of primary kidney epithelial cells, mice (postnatal day 5) were anesthetized by intraperitoneal injection of 2.5% Avertin. Kidney cortices were dissected by handheld microtome. The slices were dissociated with DMEM containing 1 mg/ml collagenase. Digested tissues were then passed sequentially through 100-μm and 45-μm sieves, and centrifuged at 1,000 × *g* for 10 min at room temperature. The pellets were resuspended and cultured in renal epithelial growth medium (REGM Bullet Kit, Cambrex Bioscience, http://www.cambrex.com).

## Supporting Information

Figure S1 Identification of *Alms1^L2131X/L2131X^* Strain (A) Genotyping data for F2 mice derived from outcross of G3 mice from an ethyl nitroso urea screen on a C57Bl/6 background to NOD, followed by intercrossing of the F1 offspring. Genotype is marked as “B” for a B6-derived allele, “H” for heterozygote, and “N” for a NOD-derived allele. Based on the phenotype and genotype data, black shading denotes the included region for the position of the mutation and gray shading denotes the excluded region. Map positions refer to public mouse genome assembly M33 (http://www.ensembl.org/Mus_musculus/index.html).
(B) Resequencing *Alms1* gene in mutant mice showing nonsense mutation at exon 10. Schematic structure of *Alms1* was drawn to scale based on a genomic search of *Alms1* cDNA (http://www.genome.ucsc.edu).
(327 KB JPG)

Figure S2 Serum Analytes in *Alms1^L2131X/L2131X^* Mice and Controls Plasma glucose, triglyceride, insulin, leptin, total cholesterol, and HDL cholesterol measurements in 60- to 80-d-old and 140- to 170-d-old *Alms1^L2131X/L2131X^, Alms1^+/L2131X^ ,* and *Alms1^+/+^* mice. Arrow, diabetic mutant male.
(445 KB JPG)

Figure S3 Loss of Cilia in the Kidney Cortex of Aged *Alms1^L2131X/L2131X^* Mice Cilia are lost in the cortex tubules (A) but not in the medulla tubules (B) in an age-dependent manner. Cilia were stained with an anti-acetylated tubulin antibody.
(2.0 MB JPG)

Table S1 Gene Expression Changes after Knockdown of *Alms1*
Gene expression levels of 98 genes with the most dramatic changes in mIMCD3 cells transfected with *Alms1* siRNAs compared to a scrambled siRNA control and a mock-transfected control.
(126 KB XLS)

### Accession Numbers

The GenBank (http://www.ncbi.nlm.nih.gov/Genbank) accession number for *Alms1* mouse cDNA is AF425257 and the RefSeq (http://www.ncbi.nlm.nih.gov/RefSeq) accession number for Alms1 mouse protein is NP660258.

## Abbreviations

ADPDK: autosomal dominant polycystic kidney disease
ARPKD: autosomal recessive polycystic kidney disease
BBS: Bardet-Biedl syndrome
IFT: intraflagellar transport
LTA: *Lotus tetragonolobus agglutinin*
mIMCD3: mouse inner medullary collecting duct
PKD: polycystic kidney disease
siRNA: short interfering RNA
